# The health system cost of post-abortion care in Rwanda

**DOI:** 10.1093/heapol/czu006

**Published:** 2014-02-17

**Authors:** Michael Vlassoff, Sabine F Musange, Ina R Kalisa, Fidele Ngabo, Felix Sayinzoga, Susheela Singh, Akinrinola Bankole

**Affiliations:** ^1^Guttmacher Institute, New York, NY, USA, ^2^School of Public Health, National University of Rwanda, P.O. Box 5229, Kigali, Rwanda and ^3^Maternal and Child Health Unit, Ministry of Health, P.O. Box 84, Kigali, Rwanda

**Keywords:** Abortion, cost, PAC, post-abortion care, Rwanda

## Abstract

Based on research conducted in 2012, we estimate the cost to the Rwandan health-care system of providing post-abortion care (PAC) due to unsafe abortions, a subject of policy importance not studied before at the national level. Thirty-nine public and private health facilities representing three levels of health care were randomly selected for data collection from key care providers and administrators for all five regions. Using an ingredients approach to costing, data were gathered on drugs, supplies, material, personnel time and hospitalization. Additionally, direct non-medical costs such as overhead and capital costs were also measured. We found that the average annual PAC cost per client, across five types of abortion complications, was $93. The total cost of PAC nationally was estimated to be $1.7 million per year, 49% of which was expended on direct non-medical costs. Satisfying all demands for PAC would raise the national cost to $2.5 million per year. PAC comprises a significant share of total expenditure in reproductive health in Rwanda. Investing more resources in provision of contraceptive services to prevent unwanted or mistimed pregnancies would likely reduce health systems costs.

**KEY MESSAGE**

- Treating complications from unsafe abortion costs $93 per case on average. Expenditure on post-abortion care is an important drain on health resources in Rwanda. Preventing unwanted pregnancies through investments in family planning would lead to net savings of scarce health resources.

## Introduction

Nineteen years after the 1994 genocide that destroyed health infrastructure and led to the loss of almost a million lives, Rwanda has made impressive progress in gradually rebuilding its health system and improving access to health services, including reproductive health services (Rusa *et al.* 2009; Basinga *et al.* 2011). Improvements in reproductive health care include better availability and quality of maternal health services and the inclusion of post-abortion care (PAC) in the services offered at public health facilities (Basinga *et al.* 2012). Over the past 10 years, the maternal mortality ratio in Rwanda declined considerably, but still remains high at 476 deaths per 100 000 live births (NISR 2012). In 2010, the maternal deaths surveillance and response system reported severe bleeding and infection as the leading causes of maternal deaths, while unsafe abortion practices were also recognized as contributing to high maternal mortality. The World Health Organization (WHO) estimated a ratio of 100 deaths per 100 000 live births in Eastern Africa in 2010 due to unsafe abortion (WHO 2011). In Rwanda, mortality due to unsafe abortion is unknown.

Until recently, abortion was legally permitted in Rwanda only when two physicians certified that it was necessary to save a pregnant woman’s life or protect her physical health. The May 2012 revision of the law extended the grounds for which abortion is authorized to include foetal abnormalities, rape, incest and forced marriage; legal abortions are still subject to court review and recommendations from at least two doctors (Republic of Rwanda 2012c). A 2009 study on abortion in Rwanda showed that the abortion rate (25 abortions per 1000 women aged 15–44 years) was lower than the rate for Eastern Africa (36) and similar to the rate of 28 for all Africa (Basinga *et al.* 2012; Sedgh *et al.* 2012).

Complications of unsafe abortion often require expensive treatment in terms of skilled personnel, surgical procedures, expensive drugs and supplies and prolonged hospital stays (Johnston *et al.* 2007; Henshaw *et al.* 2008; Vlassoff *et al.* 2008; Vlassoff *et al.* 2009a). A review of the literature, however, reveals that very little research has been conducted on the direct economic costs either to women and households or to national health systems in sub-Saharan Africa (Woog *et al.* 2007). Such estimates are not available for Rwanda. To begin to address this knowledge gap, this article estimates the cost of PAC to the national health-care system disaggregated by health-care level, region, severity of complication and cost component.

## Data and methods

This study is based on data collected from 39 health facilities in April–May 2012. The sample of facilities was randomly selected from a sample frame of 166 facilities providing PAC that was constructed in the 2009 study estimating abortion incidence care in Rwanda (Basinga *et al.* 2012). Our sample frame consisted of facilities providing different levels of care and ownership structures (public, faith-based and private). At the tertiary level of care, we selected all three referral hospitals that provide reproductive health care. Hospitals and health centers at the district level were grouped by region (Kigali city, North, West, East and South)^1^ and ownership. Within each region and ownership type, we randomly selected one district hospital and two health centers giving a total of 10 district hospitals and 20 health centers. Private facilities with an annual caseload of less than 12 post-abortion patients treated were excluded. We then grouped the remaining private facilities by type (hospital, polyclinics and clinics) and randomly selected one hospital, one polyclinic and four clinics.

Prior to commencing the research, the study obtained clearance letters from the National Ethic Committee, the National Institute of Statistics and the Ministry of Health. All potential participants were read an informed consent form before survey administration began. During the pretest of survey instruments, the research team explored the possibility of collecting costing data on PAC directly from facility records. But an investigation of several types of facilities showed that the data collection effort required for such an approach would have been too onerous and costly.

The methodology used in this study to estimate costs was developed by the Guttmacher Institute and is known as the Post-Abortion Care Costing Methodology (PACCM). PACCM adopts the ‘ingredients approach’ (Johns *et al.* 2003) and began as an adaption of the WHO’s Mother-Baby Package costing spreadsheet (Weissman *et al.* 1999). The method was first used in Nigeria (Bankole *et al.* 2007) and has subsequently been employed in costing studies in Ethiopia (Vlassoff *et al.* 2012a) and Uganda (Vlassoff *et al.* 2012b). The data collection instruments and the overall methodology of this study are similar to those of the Uganda study.

PACCM is a low-cost approach designed to yield cost estimates useful for policy analysis. Its perspective is that of the health-care system. Two questionnaires, one collecting data on drugs, supplies and materials and the other on all other inputs, gather information from key informants such as senior health-care providers knowledgeable about PAC and facility administrators. Direct cost data are gathered separately for all five main types of abortion complications—incomplete abortion, sepsis, shock, cervical/vaginal laceration and uterine laceration/perforation,^2^ following the WHO’s classification (WHO 1999).

Respondents were queried on the time required to treat each type of abortion complication, time spent in non-treatment activities, the length of the work year and salaries and benefits, for nine categories of health worker directly involved in PAC. Some benefits, such as professional allowances (duty fees, performance allowances), were omitted from the study to save effort. Our personnel cost estimates would thus somewhat underestimate the true cost of labour. Data on the cost of hospitalization were also collected. These included the proportion of cases hospitalized, the average length of stay and the estimated lodging and food costs per day.

Although costing studies of PAC have generally omitted direct non-medical costs (Vlassoff *et al.* 2008), they are a significant component of total costs.^3^ The United Nations document on global reproductive health costs reported that direct non-medical costs in 2008 were about twice as large as direct medical costs in sub-Saharan Africa (United Nations Economic and Social Council 2009). Vlassoff *et al.* (2008) found that between one-quarter and one-third of total PAC costs were non-medical in the African and Latin American regions. A recent study of PAC costs in Uganda (Vlassoff *et al.* 2012b), the first thoroughgoing attempt to measure the direct non-medical PAC costs at the national level,^4^ found that 68% of direct costs were non-medical.

In this study, respondents were queried on both capital and overhead costs. For capital costs, data were collected on infrastructure and equipment^5^ replacement costs and their useful lifetimes. Finally, data on overhead costs were gathered through the facility-based interviews in three areas: the numbers and salaries of 26 categories of non-medical support staff; 11 specific overhead expenses such as plant maintenance and utility charges; and specifics of outsourced contracts for security and cleaning. Overhead costs per case, as well as capital costs per case, were arrived at by using the proportion of PAC cases (due to induced abortions) to all cases, under the assumption that all types of treatments shared overhead and capital expenses equally.

The calculation of the annual capital cost for PAC, disaggregated by type of facility, used the following formula:

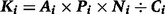

where 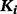 is the annual capital cost attributable to treating one PAC case, 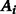 is the annual amortized cost of capital per year of useful life, 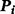 is the proportion of all cases that are PAC cases, 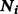 is the number of facilities in Rwanda, 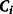 is the number of PAC cases, and 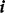 is the *i*th type of facility.

Data on the drugs, supplies and materials used in PAC were collected on over 100 inputs, but more than 400 data items were obtained to cover the dimensions of inpatient/outpatient status and type of complication. The list of drugs, supplies and lab tests was taken from previous studies in other countries and from WHO’s Mother-Baby Package (WHO 1999)*.* The prices of drugs and supplies were obtained both from the medical procurement and distribution division of the Rwanda Biomedical Center and, when local price data were lacking, from several international sources (UNFPA 2010; DrugBank 2012; IDA Foundation 2012; Management Sciences for Health 2012; Pharma Professional Services 2012; UNICEF 2012). When required, prices were adjusted to 2012 by using World Bank Gross Domestic Product (GDP) deflators (World Bank 2012). Other factors influencing final cost, either positively or negatively, such as spoilage, stock-outs, transportation costs and bulk order discounts, were not measured. The prices of laboratory tests were obtained from two private laboratories in Kigali. The cost of blood was obtained from the Rwanda blood costing model used by the National Center for Blood Transfusion of the Rwanda Biomedical Center.

Quantities of inputs reflected the amounts normally used in treating specific complications. Using these data yielded estimates of costs per treatment. As one case may involve multiple treatments, an adjustment, employing the relative shares of complication types, was necessary to calculate costs per case. A sensitivity analysis was performed in order to assess the relative effects of measurement errors on cost estimates. All variables—numbering close to 2000 in all—were tested using maximum and minimum values for each variable to compute a range of possible total cost estimates.^6^

### Estimating the number of PAC cases

The number of PAC cases in Rwanda in 2012 was estimated from the 2009 incidence of abortion study (Basinga *et al.* 2012). We obtained the number of PAC cases for 2012 by adjusting the 2009 estimate using the population growth rate (United Nations Population Division 2012) as a proxy for the growth rate in the number of PAC cases. As seen in the second panel of Table 1, three-quarters of PAC patients were treated for incomplete abortion, 13% for sepsis and 9% for shock. In general, serious complications such as lacerations and perforations were more common at higher level facilities than lower level ones. Across all 39 facilities, we found that, on average, each PAC patient was treated for just over one complication, although this ratio varied by facility type and region.

**Table 1:** Average number of PAC cases per year and distribution of abortion complications by facility type and region, Rwanda, 2012

	Referral hospitals	District hospitals	Health centers	Private clinics	Kigali	Other regions	All facilities/all regions
Average number of PAC cases per year per facility							
From incidence study (2009)^a^	484	200	37	75	100	44	57
Percent of PAC cases with specific complications							
Incomplete abortion	73.2%	70.8%	78.1%	78.9%	70.3%	79.1%	76.0%
Sepsis	6.1%	12.5%	12.8%	18.0%	17.6%	10.4%	13.0%
Shock	5.2%	14.9%	8.1%	2.5%	7.2%	9.7%	8.8%
Lacerations	4.9%	1.5%	0.0%	0.0%	1.7%	0.2%	0.8%
Perforations	10.7%	0.3%	1.0%	0.6%	3.2%	0.6%	1.5%
Total	100.0%	100.0%	100.0%	100.0%	100.0%	100.0%	100.0%
Number of PAC cases with rare complications^b^							
Number per 1000	9.3	2.5	0.1	0.0	3.0	3.0	1.4
Percent of PAC patients treated as inpatients							
Incomplete abortion	76.7%	90.0%	95.2%	21.7%	51.7%	97.6%	81.1%
Sepsis	100.0%	97.8%	98.1%	55.0%	80.6%	97.6%	91.5%
Shock	100.0%	100.0%	84.0%	99.5%	92.6%	91.2%	91.7%
Lacerations	73.3%	100.0%	100.0%	83.3%	87.1%	100.0%	95.4%
Perforations	100.0%	100.0%	100.0%	83.3%	92.9%	100.0%	97.4%
All Complications	81.6%	92.6%	94.7%	30.0%	63.5%	97.1%	83.8%
Percent of all admissions that are PAC cases^c^							
Percent of all cases	1.24%	2.76%	0.87%	0.77%	0.88%	1.32%	1.17%
Estimated number of PAC cases annually (2012) due to induced abortion							
Incomplete abortion	700	4000	7700	1500	3000	11 000	13 900
Sepsis	100	700	1200	300	900	1400	2300
Shock	100	800	800	0	500	1200	1700
Lacerations	0	100	0	0	100	0	100
Perforations	100	0	100	0	100	100	200
All complications	1000	5600	9800	1900	4600	13 700	18 300

^a^Source: Basinga *et al.* (2012).

^b^Rare complications include peritonitis, renal failure, organ failure, septicemia, poisoning, heart failure etc.

^c^‘All admissions’ means all patients who present at health facilities, both male and female.

Table 1 also shows the numbers of cases of rare but very serious complications. These include morbidities such as peritonitis, renal failure, septicemia and heart failure, which are rare but life-threatening complications. As shown in Table 1, very few such cases were reported in our study, amounting to about 1% of admissions at referral hospitals and only 0.1% of all admissions. The cost of treating these rare conditions is omitted in this study, but as each case may be very expensive, the overall cost estimates will be somewhat underestimated by this omission. In Rwanda, over four-fifths of most PAC cases were treated as inpatients in 2012. While 81% of women suffering from incomplete abortions were admitted as inpatients, 97% with uterine perforations were treated as inpatients. While around 95% of all PAC cases presenting at health centers were treated as inpatients, only 82% presenting at referral hospitals were hospitalized.

Overall, we estimated that 1.17% of all patients presenting at health facilities in Rwanda in 2012 were treated for post-abortion complications (induced or spontaneous). The number of PAC cases due to induced abortion for Rwanda in 2012 was estimated to total 18 300 (bottom panel), one-quarter in Kigali region and three-quarters in the other four regions. Even though each health center, on average, treats few PAC cases—about three per month—in total these facilities treated around 54% of all PAC cases. Many women suffering complications after unsafe abortions never reach a health facility for treatment. Using estimates from the abortion incidence study (Basinga *et al.* 2012), we estimated that in Rwanda in 2012, the number of women with *untreated* abortion complications was 9150.

### Limitations of the study

Even though our sample of health facilities was selected randomly, the small number of observations (*N* = 39) argued against the use of statistical probability analysis. Confidence intervals (not reported here) computed for the main cost components were quite wide due to the small sample. However, the sensitivity analysis does provide a range for our estimates of the total cost of treating the complications of unsafe abortion.

The data gathered through the PACCM approach are estimates made by PAC providers and facility administrators. Although the respondents have long experience in post-abortion treatments, the information they provided may suffer from unknown response biases. For instance, there may be a tendency to inflate the amount of time health workers spend with patients. The PACCM aims to keep research costs low while still generating reliable cost estimates suitable for informing policy decisions, but not managerial ones. It trades off some precision in order to minimize the time and effort in data collection.

Difficulties in measuring specific treatment inputs posed further constraints on our study. Determining the amounts of drugs and supplies used in PAC was complicated by a wide variation in nomenclature, presentation and packaging. We minimized this problem by opting for recording amounts in a few common units such as the milligram. Selecting the future rate of inflation, an important driver of capital costs, also added to imprecision. In this case, we followed the usual practice of assuming a constant rate of 3% per year. Determining the cost of labour similarly had measurement issues. Although the time spent by health workers in treatment was likely reported accurately, the proportion of total work time actually spent treating patients as opposed to time spent in non-medical activities is harder to gauge. This imprecision tended to underestimate the true cost of labour, but perhaps was compensated for by respondents inflating their estimates of the time spent in treatment.

## Results

### Direct medical costs

#### Costs of drugs, supplies, materials and laboratory tests

We estimated that the cost per PAC case attributable to unsafe abortion of drugs, supplies and other physical inputs amounted, on average, to $26 in 2012 (Table 2).^7^ Although this cost varied slightly between Kigali ($32) and other regions ($25), there were notable differences by facility type. Expenditure at district hospitals was only a little over half that at referral hospitals and health centers spent about one-quarter as much. Across all facility types, the cost of drugs and supplies was found to be considerably lower for outpatients than for inpatients (data not shown).

**Table 2:** Cost per case for PAC by facility type and region and cost per treatment by type of complication, Rwanda, 2012 (USD 2012)

Cost per case (USD)	Referral hospitals	District hospitals	Health centers	Private clinics	Kigali	Other regions	All facilities/all regions
Inputs of drugs, supplies and materials	65.38	36.55	17.25	22.34	31.82	24.58	26.26
Inputs of labour	28.01	23.22	5.39	53.40	39.85	10.22	17.07
Hospitalization	9.69	2.28	1.22	17.65	10.12	1.79	3.72
Total direct costs	103.08	62.04	23.87	93.39	81.80	36.59	47.05
Percentage distribution of costs							
Inputs of drugs, supplies and materials	63%	59%	72%	24%	39%	67%	56%
Inputs of labour	27%	37%	23%	57%	49%	28%	36%
Hospitalization	9%	4%	5%	19%	12%	5%	8%
Total direct costs	100.0%	100.0%	100.0%	100.0%	100.0%	100.0%	100.0%
**Components of cost per treatment (USD)**	**Type of complication**						
	**Incomplete abortion**	**Sepsis**	**Shock**	**Lacerations**	**Perforations**		
Inputs of drugs, supplies and materials	38.80	29.15	53.20	12.13	30.64		
Inputs of labour	15.31	30.65	38.55	15.79	19.56		
Hospitalization	3.59	8.66	5.96	1.41	5.40		

Costs of drugs and supplies by complication are shown in the bottom panel of Table 2. Shock, estimated at $53 per treatment, was the most costly complication, whereas cervical and vaginal lacerations were the least expensive ($12). The high cost of blood transfusions and the abundance of laboratory tests were the main contributors to the cost of treating shock. The main cost drivers in treating sepsis and incomplete abortions were found to be the number and frequency of laboratory tests performed such as ultrasound and blood tests. In treating uterine perforations, on the other hand, operating room supplies were the major drivers of cost.

Medicines, supplies and lab tests accounted for 56% of direct treatment costs overall. These costs represented 72% of direct medical costs in health centers, compared with around 60% in referral and district hospitals. On the other hand, they comprised only 24% of direct medical costs in private clinics, but this was largely due to the relative preponderance of less severe PAC cases in private facilities.

#### Costs of medical personnel

The percentages of PAC cases seen by the various cadres of health workers, the total time each type of worker spent in the treatment of patients and the salary and benefits received by the workers are the three main components of direct labour costs. The proportion of patients seen by specific medical cadres (data not shown) varied considerably. For example, gynaecologists or obstetricians attended 100% of patients suffering from incomplete abortion at referral hospitals, but only 53% of such patients at district hospitals (looking only at hospitals having gynaecologists or obstetricians on staff). Around 20% of incomplete abortion cases received counselling from social workers at referral and district hospitals while 54% did at health centers.

Our study found that the time spent attending patients varied by category of worker and facility, by type of complication and by region. The most notable differences were seen in private clinics where we found that health workers spent significantly more time with patients. For instance, private clinic midwives spent a total of 14 h with patients in cases of shock, whereas midwives in district hospitals spent only 3 h (data not shown). In cases of uterine perforation, private nurses spent almost 4 h tending patients but nurses at district hospitals spent only 48 min on average. Within the public system, time spent generally varied by the severity of the complication. For example, in cases of retained products of abortion, we found that on average nurses spent 50 min with patients at referral hospitals, 96 min at district hospitals and 127 min at health centers. Gynaecologists/obstetricians spent 73 min with sepsis patients at referral hospitals, but only 38 min at district hospitals having these specialists on staff.

These data, together with information on non-treatment time and salaries and benefits, allowed us to compute average costs of labour per case (Table 2, top panel). Overall, the cost of labour per PAC case was estimated to be $17 with wide variations by type of health facility, costing as much as $53 at private clinics. The higher cost in the private sector reflects greater time spent attending patients, not higher salaries, which were comparable to public sector remuneration. Within the public sector, the cost of labour for PAC was related directly to level of care, as expected, severe cases being referred to higher level facilities. Thus, labour costs $28 at referral hospitals but only $5 at health centers. Regionally, PAC labour costs were found to vary widely: on average $40 in Kigali compared with only $10 in the four rural regions. The cost of labour varied by complication (Table 2, bottom panel), but not in the way that would necessarily be expected a priori. While the average labour cost for repairing perforated uteruses was around $20, the cost of labour for treating shock was about twice as high ($39).

#### Hospitalization costs

As seen in Table 2, hospitalization (lodging and meals only) comprised only 8% of total direct medical costs overall. Hospitalization was more expensive at private clinics (around $18) than in public facilities ($1–10). Treatment of sepsis incurred the highest cost ($9) while lacerations required only $1.40 in hospitalization costs, both because relatively few laceration cases needed hospitalization and because their average length of stay was shorter than other types of complication.

The average length of stay (data not shown) varied by both type of facility and by complication. PAC patients on average spent 3.5 days in referral hospitals but only 2.2 days in health centers and 0.9 days in private clinics. In referral hospitals, patients being treated for incomplete abortion stayed, on average, for 1.7 days, 8.0 days for sepsis, 9.3 days for shock and 10.0 days for perforations.

### Direct non-medical costs

#### Overhead costs

Our study collected overhead costs in three categories: the cost of non-medical workers, maintenance/operational expenses and outsourcing costs for security and cleaning services. In 2012, the total annual wage bill for non-medical workers per facility ranged from $366 000 for referral hospitals to $39 000 for health centers (Table 3). Referral hospitals employed an average of 62 non-medical workers, district hospitals 35 workers, health centers 15 workers and private clinics 11 for ancillary, non-treatment activities. The annual cost per facility for maintenance expenses ranged from $1.9 million for referral hospitals to $43 000 for health centers. These expenses were almost three times higher for facilities in the Kigali region than in the other four regions. Outsourced contracts for cleaning and/or security cost referral hospitals $367 000 on average, but only $87 000 at district hospitals. Adding up these three components of overhead, the total average overhead cost per facility amounted to $163 000 per annum. The portion attributable to PAC (due to induced abortion) averaged $1300 per facility—$20 700 for referral hospitals, $3800 for district hospitals, $800 for health centers and $1300 for private facilities. Overhead per PAC case amounted to $28 at district hospitals, $63 at referral hospitals and $33 at health centers.

**Table 3:** Overhead and capital components of PAC cost per case by facility type and by region, Rwanda, 2012 (USD 2012)

	Referral hospitals	District hospitals	Health centers	Private clinics	Kigali	Other regions	All facilities/all regions
Overhead costs							
Annual wage bill for non-medical workers per facility^a^	366 000	219 000	39000	126 000	125 000	54 000	64 000
Annual maintenance and other operational expenses per facility^b^	1 931 000	323 000	43 000	109 000	199 000	67 000	85 000
Annual cost of outsourced contracts per facility^c^	367 000	87 000	4000	0	50 000	8000	14 000
Total annual overhead expenditure per facility	2 664 000	629 000	86 000	235 000	374 000	129 000	163 000
Total annual overhead cost to supply PAC due to induced abortion per facility	20 700	3800	800	1300	3600	900	1300
Overhead cost per PAC case (due to induced abortion)	63.14	27.66	33.03	25.13	54.06	25.60	32.18
Capital costs							
Infrastructure and equipment costs per facility							
Amount per facility	66 560 000	3 570 000	1 490 000	1 710 000	15 890 000	2 120 000	7 060 000
Useful lifetime							
Number of years	40	40	42	50	46	40	42
Amortized annual infrastructure and equipment costs to supply PAC due to induced abortion^d^							
Amount per facility	23 800	500	500	400	5500	400	2300
Capital cost per PAC case (due to induced abortion)	72.55	3.16	14.92	6.67	25.61	9.95	13.57

^a^Data were collected on 26 categories of non-medical workers such as guard, cleaner, receptionist, record keeper, supply clerk, etc.

^b^Data were collected on 11 operational expenses such as building maintenance, utilities, vehicle maintenance, travel expenses, audio/visual materials, education/reference materials, etc.

^c^Cleaning and security.

^d^The future annual rate of inflation was assumed to be 3%.

#### Capital costs

Capital costs were estimated from survey data on the costs of the facilities’ infrastructure and equipment as well as data on the useful lifetimes of the facilities. Referral hospitals, as expected, were the most expensive, costing $66.6 million on average to construct and equip, while health centers were the least costly at $1.5 million per facility. Regionally, capital spending per facility in the Kigali region was more than seven times that in the rest of the country. The estimated useful lifetimes of facilities varied little among public and faith-based facilities (40–42 years), while private facilities had an estimated lifetime of 50 years. Overall amortization costs for PAC due to unsafe abortion were found to be $2300 per facility, ranging from $23 800 for referral hospitals to $400–500 for other types of facilities. The cost of capital was greatest for referral hospitals at $73 per PAC case and least at district hospitals at $3 per PAC case. The cost of capital per PAC case in Kigali was estimated to be more than 2.5 times higher than in the four surrounding regions.

#### Total direct costs per case

Table 4 displays the total direct costs of all inputs per PAC case, both medical and non-medical, by facility type and by region, aggregated across all complication types. The average cost per PAC case due to unsafe abortion was estimated to be $93 and was directly related to the level of care: treatment of post-abortion cases at referral hospitals was the most costly ($239 on average); per-case PAC treatment at district hospitals cost $93, while $72 was spent per case at health centers.

**TABLE 4:** Cost per case PAC due to induced abortion by facility type and region, Rwanda, 2012 (USD 2010)

Components of cost	Referral hospitals	District hospitals	Health centers	Private clinics	Kigali	Other regions	All facilities/all regions
Direct medical costs^a^	103.08	62.04	23.87	93.39	81.80	36.59	47.05
Direct non-medical costs^b^							
Overhead	63.14	27.66	33.03	25.13	54.06	25.60	32.18
Capital	72.55	3.16	14.92	6.67	25.61	9.95	13.57
Total direct non-medical costs	135.69	30.82	47.95	31.80	79.67	35.55	45.76
Total cost per PAC case	238.77	92.86	71.81	125.19	161.47	72.14	92.81
Percentage distribution							
Direct costs	43.2%	66.8%	33.2%	74.6%	50.7%	50.7%	50.7%
Indirect costs	56.8%	33.2%	66.8%	25.4%	49.3%	49.3%	49.3%
Total cost per PAC case	100.0%	100.0%	100.0%	100.0%	100.0%	100.0%	100.0%

^a^From Table 2.

^b^From Table 3.

We found that non-medical expenditures on capital and overhead, necessary for the functioning of the health system although not directly tied to the provision of care, were 51% of total cost per case. This non-medical component ranged from 67% of total cost at health centers to 33% at district hospitals.

## Discussion

### National cost of PAC due to unsafe abortion

Our results on cost per case and cost per treatment of PAC were extended to compute the full monetary impact of unsafe abortion on the national health system. From our data and from the findings of the 2009 abortion incidence study (Basinga *et al.* 2012), we were able to disaggregate the estimated 18 300 PAC cases in 2012 caused by induced abortions by type of complication, facility type and region. Applying these numbers to the relevant costs per case yielded national cost totals (Table 5). The bottom panel of Table 5 displays the total costs to the Rwanda health system for treating PAC in 2012 by facility type and region. Including direct medical and non-medical costs, the grand total for Rwanda was estimated to be $1.7 million. A little under half of this amount, or $837 000, went to non-medical costs (overhead and infrastructure) while the remainder ($861 000) was spent on direct medical inputs (drugs, supplies, labour and hospitalization). Hospitalization accounted for only 4% of total expenditure, drugs and supplies for 28% and the wages of medical personnel for 18%.

**Table 5:** Estimates of total annual costs at the national level of PAC (due to induced abortion) by facility level, region and type of complication, Rwanda, 2012 (USD 2012)

Cost components	Referral hospitals	District hospitals	Health centers	Private clinics	Kigali	Other regions	All facilities/all regions
National cost of labour for PAC (USD)							
All complications	28 000	130 000	53 000	102 000	169 000	144 000	312 000
National cost of drugs, supplies and materials for PAC (USD)							
All complications	64 000	204 000	169 000	43 000	135 000	346 000	481 000
National cost of hospitalization for PAC (USD)							
All complications	10 000	13 000	12 000	34 000	43 000	25 000	68 000
National infrastructure and equipment cost to supply PAC (USD)							
Capital cost to system to supply PAC	71 000	18 000	147 000	13 000	108 000	140 000	248 000
National overhead cost to supply PAC (USD)							
Overhead cost to system to supply PAC	62 000	154 000	324 000	48 000	229 000	360 000	589 000
Total national cost of PAC (USD)	235 000	519 000	705 000	240 000	684 000	1 015 000	1 698 000
Total direct cost of PAC	102 000	347 000	234 000	179 000	347 000	515 000	861 000
Total indirect cost of PAC	133 000	172 000	471 000	61 000	337 000	500 000	837 000

Treating incomplete abortion accounted for the largest share of total expenditure on PAC: 52% of labour costs, 80% of the cost of drugs and supplies and 66% of hospitalization costs were spent in treating this complication (data not shown). Treatment of sepsis and shock accounted for most of the remainder of direct medical costs, while lacerations and perforations together consumed only around 2–4% of direct medical inputs. Most PAC expenditure occurred in health centers ($705 000) followed by district hospitals ($519 000). Referral hospitals accounted for $235 000 or only 14% of total expenditure on PAC caused by unsafe abortion.

### Sensitivity analysis

As the small size of our sample precluded the use of statistical inference to obtain confidence intervals, we undertook a sensitivity analysis to gain an understanding of the extent to which imprecisely measured inputs would affect cost estimates. Data on several hundred inputs were collected in the study. We attempted to test the sensitivity of most, focusing on their impact on total national cost.

Some results of this analysis are shown in Table 6. The top panel displays the 10 inputs (not related to drugs or supplies) that had the largest impacts. We found that cost estimates were most sensitive to variations in capital and overhead data as well as some labour inputs. The three inputs determining capital costs were ranked first, third, and fifth in terms of sensitivity. For example, raising the data values for the variable ‘infrastructure and equipment cost’ by 25% raised the total national cost estimate by 4.02%. In terms of sensitivity to overhead inputs, transportation, utilities, building maintenance and outsourced service contracts were the biggest drivers of total PAC cost. Three labour-related inputs also ranked among the top 10 most sensitive variables: the numbers of hours in a work year for nurses/midwives and doctors and the salaries of midwives. As can be seen, the tenth most significant input had an impact of only 0.74% on total cost in the sensitivity test. Of 219 non-drug/supply inputs tested, 157 had an impact of less than 0.05% on the total cost of PAC.

**Table 6:** Results of sensitivity analysis: the variables to which national total PAC cost is most sensitive, Rwanda, 2012

Cost input	Cost component contributed to	Absolute difference between minimum and maximum (USD)	Percentage change in national total cost
Labour, hospitalization, overhead, capital			
Infrastructure and equipment cost	Capital	124 200	4.02
Number of hours in work year (nurses/midwives)	Labour	73 500	2.38
Average lifetime of facility	Capital	68 100	2.21
Number of hours in work year (doctors)	Labour	59 700	1.94
Future rate of inflation	Capital	59 700	1.93
Transport^a^	Overhead	31 000	1.00
Salary of midwives	Labour	30 000	0.97
Cost of outsources service contracts	Overhead	28 800	0.93
Maintenance and operational costs	Overhead	25 800	0.84
Utilities^b^	Overhead	22 700	0.74
Drugs, supplies, lab tests			
Amoxicillin	Drugs/supplies	19 600	0.58
Test for syphilis	Drugs/supplies	18 300	0.54
Test for HIV	Drugs/supplies	18 000	0.53
Test: complete blood count (NFS)^c^	Drugs/supplies	17 500	0.52
Metronidazole by injection	Drugs/supplies	16 600	0.49
Ultrasound	Drugs/supplies	13 700	0.40
Ringer lactate (Hartmann's solution)	Drugs/supplies	13 600	0.40
Urine cyto-bacteriological test (ECBU)^d^	Drugs/supplies	10 800	0.32
Blood for transfusion (500 ml bag)	Drugs/supplies	9600	0.28
Pregnancy test	Drugs/supplies	8600	0.25

^a^Fuel, repairs, maintenance, lodging, etc.

^b^Water, gas, electricity, telephone, waste collection, etc.

^c^In French: Numeration formule sanguine (NFS).

^d^In French: Examen Cyto-Bacteriologique des Urines (ECBU).

The lower panel of Table 6 shows the 10 inputs of drugs, supplies and materials—including lab tests—whose variation influenced total cost the most. Six inputs in this top 10 list were either laboratory tests or the abdominal ultrasound procedure. The test for syphilis, for example, caused a 0.54% change in total cost when its data values were either raised or lowered by 25%. Of the 521 physical inputs tested, only 22 changed total cost by 0.1% or more and 411 inputs had a negligible effect (0.01% or less).

### Rwanda and Uganda: contrasting PAC costs of two neighbours

As mentioned, the current study closely mirrors a 2010 costing study in Uganda, both in methodology and in proximity in time, making it interesting to explore the similarities and differences between the responses of these neighbouring countries’ health systems to the challenges thrown up by unsafe abortion.

Table 7 lists several indicators used for this comparison. We note, first of all, that income levels and overall spending on health were broadly similar in the two countries. The cost of treating post-abortion complications from unsafe abortion was similar in the two countries: $93 per case in Rwanda and $138 in Uganda. Breaking down the total cost figure into its major components (Table 7, second panel), we see further similarities: drugs/supplies, labour and hospitalization costs were roughly comparable between countries. On the other hand, the overhead component in Rwanda ($32) was 60% higher than in Uganda ($20), while capital costs in Rwanda were only one-fifth as large as Uganda’s. Medical salaries were substantially higher in Rwanda than in Uganda (lower panel). The amount of time medical cadres spent with PAC patients was also higher in Rwanda than Uganda, further adding to the gap in per-case costs. Table 7 also shows two examples of material inputs: blood sacs for transfusions and haemoglobin tests. In both cases, Rwandan prices were above Ugandan ones.

**Table 7:** Comparing the cost of PAC in Rwanda and Uganda, 2012

	Uganda	Rwanda
General indicators		
Total population	36 269 000	11 460 000
Number of women aged 15–44	7 351 000	2 589 000
Number of PAC cases^a^	113 000	18 300
Number of PAC cases per 1000 woman aged 15–44	15.37	7.07
Gross domestic product per capita^b^	$1345	$1282
Health expenditure per capita (USD, 2010)	$46.72	$55.51
Reproductive health expenditure per capita (USD)^c^	$9.65	$3.61
PAC expenditure per capita (USD)	$0.38	$0.15
Cost per case of PAC (USD)	$138.17	$92.81
Components of cost of PAC		
Cost of labour per PAC case (USD)	$11.75	$17.07
Cost of drugs/supplies per PAC case (USD)	$25.46	$26.26
Cost of hospitalization per PAC case (USD)	$5.90	$3.72
Cost of capital per PAC case (USD)	$74.66	$13.57
Cost of overhead per PAC case (USD)	$19.89	$32.18
Specific cost-related indicators		
Percent of PAC cases treated at:		
Primary level	64%	60%
Secondary level	28%	34%
Tertiary level	7%	6%
Percent of PAC cases treated for:		
Incomplete abortion	62%	76%
Shock	21%	13%
Sepsis	9%	9%
Lacerations or perforations	9%	2%
Average monthly salary (USD):		
Obstetrician/gynaecologist	$592	$946
Nurse-midwife	$219	$401
Pharmacist	$349	$434
Average time spent, doctor, sepsis (min)	71	53
Average time spent, nurse, sepsis (min)	88	131
Average time spent, doctor, incomplete abortion (min)	49	46
Average time spent, nurse, incomplete abortion (min)	88	128
Cost of one unit of blood (USD)	$7.79	$48.60
Cost of haemoglobin test (USD)	$0.85	$1.32

Note: Unless otherwise stated, Ugandan costs inflated by US inflation rate for period 2010–12.

Sources: World Bank, United Nations Population Division, US Bureau of Labor, Vlassoff *et al.* (2012b), data from this study.

^a^PAC cases due to induced abortions.

^b^Purchasing power parity (PPP), current international dollars, 2011.

^c^For Uganda, annual expenditure for maternal and newborn health, 2010.

The distribution of patients by level of health care was quite similar in the two countries. However, the distribution of PAC cases by type of complication showed that Uganda had a somewhat heavier burden of serious cases. This likely indicates that access to safe—though still clandestine—abortions was probably greater in Rwanda than in Uganda.

## Conclusion and implications

We estimate the cost of treating the consequences of unsafe abortion to the Rwanda health system in 2012 at $1.7 million, non-medical costs (overhead and infrastructure) comprising 49% of this total. Improving ‘accessibility to quality, and demand for, maternal and child health services’ is one of the strategic objectives of the Rwanda health sector strategic plan (Republic of Rwanda 2012b). This increased government prioritization towards reproductive health services is reflected by the 116% increase in reproductive health total expenditures in 2009–2010 compared with 2006, with an increase of the publicly funded share of reproductive health expenditures from 14% to 38% (Republic of Rwanda 2012b). Total expenditure on health in Rwanda in 2009–2010 was estimated at $420.3 million and, of this amount around 10%—$41.4 million—were allocated to reproductive health services (Republic of Rwanda 2012a). Thus, our estimates show that the cost of treating post-abortion complications from unsafe procedures was 4.1% of projected spending on reproductive health and 10.6% of total public spending on reproductive health. The cost of unsafe abortion to the health system is therefore a considerable one.

According to Basinga *et al.* (2012), almost one-third of women who experienced post-abortion complications did not receive treatment. If all women with complications had been able to access health-care services, the total cost of PAC would have increased to $2.5 million in 2012. However, we should note that our estimates are based on expert opinion, not population-based data, and also that women not attending health facilities may have less severe symptoms on average than those who do attend. Despite this uncertainty, there is no doubt that treating all women who have an unmet demand for PAC would represent a sizable additional financial burden on scarce health resources, should this need be met.

Unsafe abortion generates unnecessary costs to society on a variety of levels (Vlassoff *et al.* 2008). This study focuses on only one component of the cost of unsafe abortion—the cost to the health-care system of treating post-abortion complications. There are many other substantial costs involved (Vlassoff *et al.* 2008) including the treatment of longer term morbidities that result from unsafe abortion—especially the high cost of infertility treatment—as well as the economic cost to Rwandan households and society of productive time lost through abortion-related morbidity and mortality. The total financial burden of unsafe abortion would therefore be much higher than the costs we are able to report from our study.

Preventing the root causes of unsafe abortion is essential. Basinga *et al.* (2012) estimated that 47% of all pregnancies (about 275 000 in all) were unintended. The substantial cost estimates from our study reinforce the importance of reducing unsafe abortion by increasing contraceptive coverage and PAC in a country where abortion is restricted. A better health policy would address this root cause of unsafe abortion by addressing the large unmet need for contraception which still stood at around 19% in 2010, having declined from 38% in 2005 (NISR 2009, 2012). Cost–benefit comparisons in Nigeria (Bankole *et al.* 2007), Ethiopia (Vlassoff *et al.* 2012a) and Uganda (Vlassoff *et al.* 2012b) demonstrated that more spending on family planning would achieve substantial savings from reduced expenditure in PAC. Although no recent data are available on the cost of family planning services in Rwanda, if we use costs of contraceptive services found in neighbouring countries (around $30 per user)^8^ as a proxy, we can make a similar comparison for Rwanda. Comparing this cost to the per-case cost of PAC ($93), it is clear that there exists a strong economic rationale for preventing unwanted pregnancies in Rwanda through family planning. The marginal benefit–cost ratio of this strategy would be more than 3:1; that is, every franc spent in family planning would save the health system more than three francs in PAC treatments averted.

Rwanda has achieved remarkable health gains over the last decade; the cost estimates from this study provide evidence to further improve health policies and programmes to continue the positive trend towards increasing contraceptive use and access to PAC.

## Supplementary Data

Supplementary data are available at *Health Policy and Planning* online.

Translated Abstracts

## Footnotes

^1^ Although our sample was based on Rwanda’s five regions, four regions (North, East, South and West) are largely rural in character whereas the fifth region, Kigali, is largely urban. Since findings from the four rural regions were very similar, in the analysis of the data we opted to contrast Kigali with the other four regions taken together.

^2^ The category ‘uterine perforation’ includes cases of other lower abdominal perforations as well as hysterectomies.

^3^ We use the term ‘direct non-medical costs’ to refer to overhead and capital costs to maintain consistency with a similar study in Uganda (Vlassoff *et al.* 2012b). We note that these costs are often, though not universally, called ‘indirect costs’.

^4^ A thorough study of all cost components of four facilities in Mexico City (Levin *et al.* 2009) found comparably high direct non-medical costs.

^5^ ‘Equipment’ included all medical mechanical devices, furniture and test equipment as well as vehicles.

^6^ For all variables, the minimum was set at 75% of the mean value and the maximum at 125% of the mean.

^7^ All costs in this study are given in 2012 United States Dollars (USD).

^8^ The average annual costs of family planning per user are as follows: Ethiopia $32.21 (Sundaram *et al.* 2010), Uganda $22.24 (Vlassoff *et al.* 2009b) and Burkina Faso $34.75 (Vlassoff *et al.* 2011).
