# Supplementary material for: The health system cost of post-abortion care in Rwanda
Source: Health Policy Plan. 2014 Feb 17;30(2):223–33. doi: 10.1093/heapol/czu006 (PMC4325535; doi:10.1093/heapol/czu006)
Supplement: Translated Abstracts [file supp_30_2_223__index.html]

The health system cost of post-abortion care in Rwanda — The health system cost of post-abortion care in Rwanda — Translated Abstracts 

# The health system cost of post-abortion care in Rwanda

## Translated Abstracts

files

**Files in this Data Supplement:**

- Chinese Abstract - pdf file
- French Abstract - pdf file
- Spanish Abstract - pdf file
