# Supplementary material for: The health system cost of post-abortion care in Rwanda
Source: Health Policy Plan. 2014 Feb 17;30(2):223–33. doi: 10.1093/heapol/czu006 (PMC4325535; doi:10.1093/heapol/czu006)
Supplement: Translated Abstracts [file supp_czu006_czu006_Chinese.pdf]

卢旺达堕胎后医疗服务的医疗体系成本研究

我们计划根据 2012 年的研究结果来估计卢旺达医疗系统为由于不安全堕胎而提供的堕胎后医疗服务的成本，这是一个没有在国家层面上进行过研究的重要政策课题。在全国的所有五个地区，我们随机选出了能够代表三个医疗层面的 39 个公共和私立医疗机构，从关键服务提供者和管理者那里收集数据。使用要素构成法来计算成本，我们的数据从药品、供应、材料、人员时间以及住院治疗这几个方面进行收集。此外，直接的非药物成本也被计算进来，比如管理费用和资本成本。我们发现，每个客户的平均年堕胎后医疗成本为 93 美元，其中包括了 5 种堕胎后的并发症。全国每年堕胎后医疗的成本估计为一千七百万美元，其中有 47%都被用在了直接的非药物费用上。如果要满足所有的医疗服务需求，成本将上升到每年两千五百万美元。在卢旺达，堕胎后医疗服务的支出占据了总的生殖医疗支出的很大一部分。因此，在避孕方面进行更多的投资来避免不需要的怀孕和时机不合适的怀孕可以有效得减少医疗支出成本。
