# Supplementary material for: The health system cost of post-abortion care in Rwanda
Source: Health Policy Plan. 2014 Feb 17;30(2):223–33. doi: 10.1093/heapol/czu006 (PMC4325535; doi:10.1093/heapol/czu006)
Supplement: Translated Abstracts [file supp_czu006_czu006_French.pdf]

Coûts des soins après avortement pour le système de santé au Rwanda

Accepté le 7 janvier 2014

En se basant sur des études menées en 2012 au Rwanda, nous avons estimé le coût des soins après avortement (SAA) pour le système de santé à la suite d'avortement à risque, un objectif politique important qui n'a pas encore été étudié au niveau national. Trente-neuf établissements de santé, publics et privés représentant trois niveaux de soins pour les cinq régions du Rwanda, ont été sélectionnés au hasard afin de collecter des données auprès des principaux professionnels de la santé et des administrateurs. En utilisant un modèle d'estimation des coûts fondé sur une approche par élément, nous avons collecté des données sur les médicaments, l'approvisionnement, le matériel, le temps du personnel et les hospitalisations.
Nous avons, en plus, calculé les coûts non médicaux directs tels que les frais généraux et les coûts d'exploitation. Nous avons pu calculer que le coût moyen des SAA par patiente est de $93 et ceci intégrant cinq différents types de complications au cours d'un avortement. Le coût total des SAA au niveau national est estimé à 1,7 million de dollars US par an, dont 49% ont été dépensés en coûts non médicaux directs. Afin de satisfaire toute la demande de SAA, il faudrait que ces coûts soient d'environ 2,5 millions de dollars US par an. Les SAA participent pour une part importante aux dépenses totales de la santé reproductive au Rwanda. Mettre plus de ressources dans les moyens de contraception afin d'empêcher les grossesses inopportunes et non désirées permettrait de réduire les coûts pour le système de santé.

Mots Clés : Avortement, coût, SAA, soins après avortement, Rwanda
