# Supplementary material for: The health system cost of post-abortion care in Rwanda
Source: Health Policy Plan. 2014 Feb 17;30(2):223–33. doi: 10.1093/heapol/czu006 (PMC4325535; doi:10.1093/heapol/czu006)
Supplement: Translated Abstracts [file supp_czu006_czu006_Spanish.pdf]

El costo al sistema de salud de la atención post-aborto en Ruanda

Michael Vlassoff, Sabine F. Musange, Ina R. Kalisa, Fidele Ngabo, Felix Sayinzoga, Susheela Singh y Akinrinola Bankole

Aceptado 7 de enero de 2014

Con base en la investigación realizada en 2012, se estima el costo al sistema de salud de Ruanda de brindar atención post-aborto (APA) como resultado de abortos inseguros, un tema de importancia para la política que no se ha estudiado antes a nivel nacional. Treinta y nueve instalaciones de salud públicas y privadas que representan tres niveles de atención de salud fueron seleccionadas al azar para la recolección de datos de proveedores y administradores de atención claves en las cinco regiones. Utilizando un enfoque de ingredientes para el cálculo de costos, se recopilaron datos sobre medicamentos, suministros, material, tiempo del personal y hospitalización. Adicionalmente, se midieron también los costos no médicos directos como los gastos generales y los costes de capital. Se encontró que el costo anual promedio de la APA por cliente, a lo largo de cinco tipos de complicaciones del aborto, era $93. El coste total de la APA a nivel nacional se estimó en $ 1,7 millones al año, el 49% de los cuales se gastó en los costos no médicos directos. Satisfacer todas las demandas de la APA elevaría el costo nacional a $2,5 millones por año. La APA comprende una parte significativa del gasto total en salud reproductiva en Ruanda. Invertir más recursos en la prestación de servicios de anticoncepción para evitar embarazos no deseados o inoportunos probablemente reduciría los costos a los sistemas de salud.

Palabras Claves
Aborto, costo, APA, atención post-aborto, Ruanda

MENSAJE CLAVE

- El tratamiento de las complicaciones como resultado del aborto inseguro cuesta $93 por caso, en promedio. El gasto en la atención post-aborto es un drenaje importante de los recursos de salud en Ruanda. La prevención de los embarazos no deseados a través de las inversiones en planificación familiar daría lugar a un ahorro neto de recursos de salud escasos.
